# Hydrometeorological conditions drive long-term changes in the spatial distribution of *Potamogeton crispus* in a subtropical lake

**DOI:** 10.3389/fpls.2024.1424300

**Published:** 2024-07-09

**Authors:** Ke Yang, Yi Yin, Ying Xu, Shaobo Wang, Mingyuan Gao, Kai Peng, Juhua Luo, Junfeng Gao, Yongjiu Cai

**Affiliations:** ^1^ Key Laboratory of Lake and Watershed Science for Water Security, Nanjing Institute of Geography and Limnology, Chinese Academy of Sciences, Nanjing, China; ^2^ University of Chinese Academy of Sciences, Beijing, China; ^3^ Jiangsu Surveying and Design Institute of Water Resources Co., Ltd., Yangzhou, China; ^4^ Jiangsu Province Hydrology and Water Resources Investigation Bureau, Nanjing, China

**Keywords:** *Potamogeton crispus*, spatiotemporal pattern, climate change, hydrometeorology, water quality, shallow lakes

## Abstract

Globally, anthropogenic disturbance and climate change caused a rapid decline of submerged macrophytes in lake ecosystems. *Potamogeton crispus* (*P. crispus*), a species that germinates in winter, explosively expanded throughout many Chinese lakes, yet the underlying mechanism remained unclear. Here, this study examined the long-term changes in the distribution patterns of *P. crispus* in Lake Gaoyou by combining remote sensing images and hydrometeorological data from 1984 to 2022 and water quality data from 2009 to 2022. It aims to unravel the relationships between the distribution patterns of *P. crispus* and hydrometeorological and water quality factors. The results showed that the area of *P. crispus* in Lake Gaoyou showed a slight increase from 1984 to 2009, a marked increase from 2010 to 2019, followed by a decline after 2020. Spatially, *P. crispus* was primarily distributed in the western and northern parts of Lake Gaoyou, with less distribution in the central and southeastern parts of the lake. Wind speed (WS), temperature (Temp), water level (WL), ammonia nitrogen (NH_3_-N), and Secchi depth (SD) were identified as the key factors regulating the variation in the *P. crispus* area in Lake Gaoyou. We found that the *P. crispus* area showed an increasing trend with increasing Temp, WL, and SD and decreasing WS and NH_3_-N. The influence of environmental factors on the area of *P. crispus* in Lake Gaoyou varied among seasons. The results indicated that hydrometeorology (WS, Temp, and WL) may override water quality (NH_3_-N and SD) in driving the succession of *P. crispus* distribution. The findings of this study offer valuable insights into the recent widespread expansion of *P. crispus* in shallow lakes across Eastern China.

## 1 Introduction

As a crucial producer in aquatic ecosystems, macrophytes not only prevent sediment resuspension, reduce nutrient release, and inhibit the growth of phytoplankton, but also provide habitats for various aquatic organisms (Søndergaard et al., 2010). As one of the important types of macrophyte, submerged macrophytes play an important role in ecological restoration; the water body in which they are present typically has low nutrient levels and phytoplankton biomass (Sayer et al., 2010). Generally, submerged macrophytes absorb nutrients from the water body during their growth period, significantly improving water quality and increasing transparency (Wu and Hua, 2014). However, extensive submerged macrophyte decomposition following excessive growth can have negative impacts on aquatic ecosystems by depleting dissolved oxygen (DO) in the water, which is an important cause of water quality degradation in macrophyte-dominated eutrophic lakes (Roman et al., 2001; Wang et al., 2018). Therefore, maintaining an appropriate level of submerged macrophyte coverage is essential for maintaining water quality and aquatic ecosystem stability, as well as for ecological restoration (Temmink et al., 2021).

Over the past few decades, many lake ecosystems in China have undergone rapid degradation, characterized mainly by reduction in macrophyte coverage, dominance of single species, and overgrowth of specific species (Cao et al., 2015; Dong et al., 2022). These phenomena were influenced by various factors, including global climate change and anthropogenic activities. Previous studies have indicated that an increase in temperature promotes the germination of macrophytes, affecting their reproductive strategies, interactions, and species richness (Zhang YL et al., 2016; Li et al., 2017; Fares et al., 2020; Kim and Nishihiro, 2020). Changes in hydrological conditions such as water level and flow velocity are important factors contributing to the significant decrease in submerged macrophyte coverage and diversity (Breugnot et al., 2008; Luo et al., 2015). Anthropogenic activities may cause eutrophication in lakes and trigger a shift from macrophyte-dominated to phytoplankton-dominated states; a regime shift theory had been developed to describe the abrupt changes (Scheffer et al., 1993; Akasaka et al., 2010; Zhang PY et al., 2016). Previous studies have explored the impact of environmental factors on submerged macrophyte decline, investigating the spatiotemporal variability of the distribution of submerged macrophyte in eastern lakes such as Lake Taihu in China through field investigation, controlled experiment, remote sensing, and ecological modeling (Zhang YL et al., 2016; Dong et al., 2022). These studies mainly focused on the decline of macrophyte coverage. However, studies on the explosive growth of single species are also necessary; in recent years, *Potamogeton crispus* (*P. crispus*) has expanded explosively in many shallow lakes in China and disrupted water quality and the stability of aquatic ecosystems (Cao et al., 2015; Huang et al., 2022).

*P. crispus* is a submerged macrophyte that requires lower temperatures during its growth period; it usually germinates in the winter, grows in the spring, and then degrade in late spring and early summer (Jian et al., 2003; Woolf and Madsen, 2003). Because of this unique phenological character, *P. crispus* has emerged as a predominant submerged macrophyte species in most shallow lakes in Eastern China in spring (Cao et al., 2015; Chen et al., 2017). Among these, there were few submerged macrophyte species in Lake Gaoyou, and *P. crispus* has emerged as a dominant species in recent years; it spread across the entire lake during spring (Tian et al., 2019; Xia et al., 2022). Following its bloom period, *P. crispus* decomposes quickly, releasing nutrients that have a substantial negative influence on water quality and the stability of aquatic ecosystems, potentially endangering the safety of the local water supply security (Wang et al., 2018; Huang et al., 2022). Therefore, elucidating the factors influencing the growth of *P. crispus* appears to be quite important. Since the 1990s, there have been field investigations on macrophyte communities in some lakes, such as Lake Nansi and Lake Dongping (Yu et al., 2017; Xia et al., 2022). In recent years, some studies used remote sensing technologies to interpret and identify wetlands and macrophyte distribution (Wang et al., 2019; Huang et al., 2021). However, there have been few studies on the long-term spatiotemporal variability of the distribution and driving factors of *P. crispus*.

Here, this study combined remote sensing images and hydrometeorological factors of Lake Gaoyou from 1984 to 2022 and water quality factors from 2009 to 2022 to achieve the following research objectives: (1) clarify the long-term changes in distribution characteristics of *P. crispus* in Lake Gaoyou, and (2) disentangle the relative importance of hydrometeorology and water quality factors in regulating the area of *P. crispus* in Lake Gaoyou. We hypothesized that long-term changes in distribution patterns of *P. crispus* were strongly related to hydrometeorological conditions compared to water quality because of climate change in the past decades (Wu et al., 2021; Xia et al., 2022). This study could provide insights into understanding the mechanisms behind the recent large-scale blooms of *P. crispus* in shallow lakes of Eastern China.

## 2 Materials and methods

### 2.1 Study area

**Figure 1:**
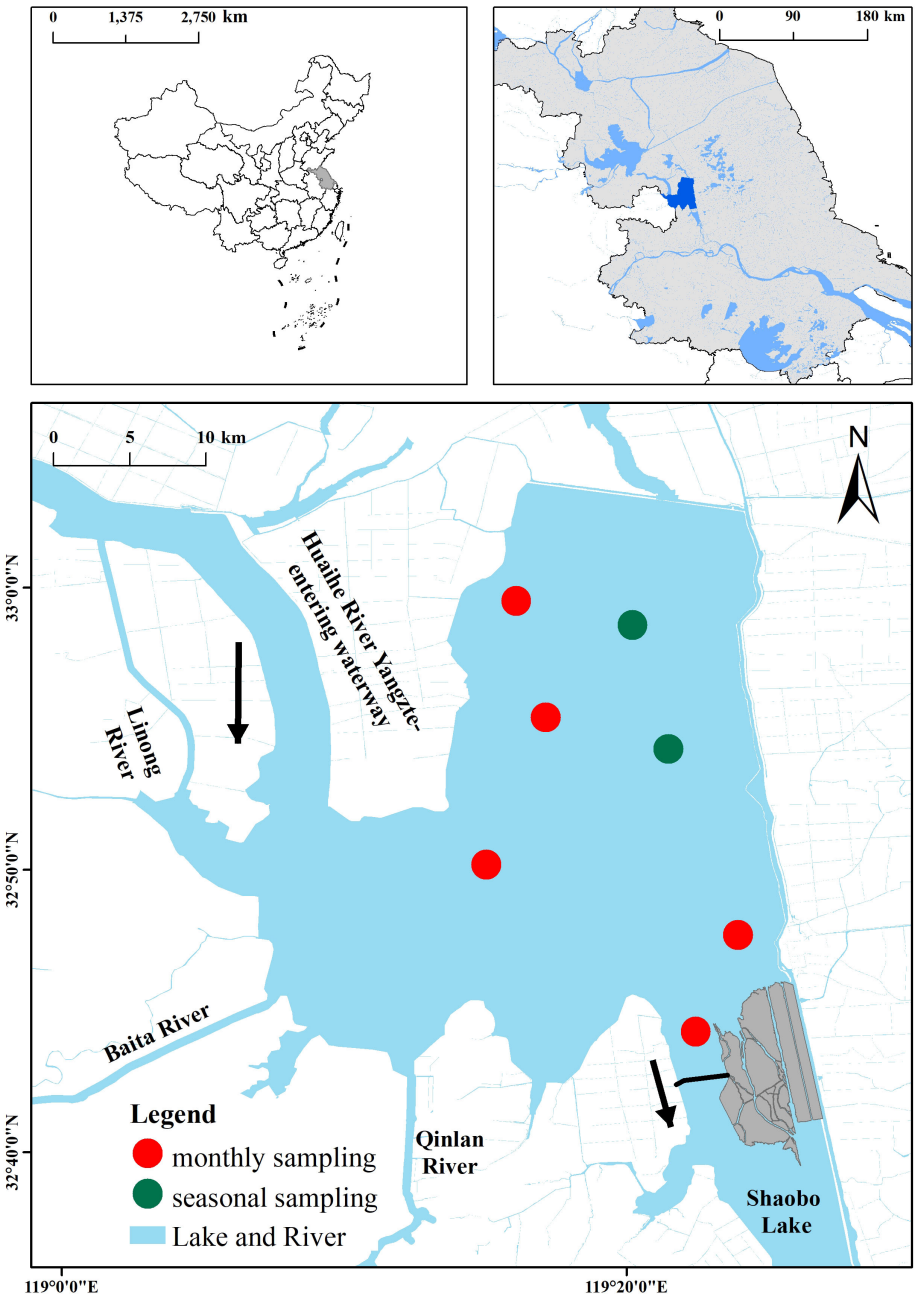
Map showing Lake Gaoyou and the distribution of sampling sites. Red circles indicate the monthly sampling sites and green circles indicate the seasonal sampling sites.

Lake Gaoyou (32°30′–33°05′N, 119°06′–119°25′E) is located in the central part of Jiangsu Province, China, in the downstream area of the Huai River; it mainly receives water from the Huai River (**Figure 1**). The total area of the water body is 728 km^2^. Lake Gaoyou is situated in the subtropical monsoon climate zone, with an average annual precipitation of 1,029 mm and an average annual evaporation of 890 mm (Chen et al., 2017). The prevailing wind direction is southeast. The main rivers along the lake include Linong River, Baita River, and Qinlan River. Lake Gaoyou is a typical overflow lake, primarily playing a crucial role in flood control and water supply. More importantly, it serves as a water source for the Eastern Route of the South-to-North Water Diversion Project (ER-SNWDP), thus contributing to water diversion benefits and drinking water safety (Qu et al., 2020). However, Lake Gaoyou is undergoing drastic changes in hydrological regime and is strongly impacted by anthropogenic activities such as reclamation and enclosed aquaculture, leading to eutrophication (Guo et al., 2023).

### 2.2 Remote sensing data collection and analysis

The remote sensing data for this study are Landsat 5 and Landsat 8 satellite data, with a spatial resolution of 30 m. The Landsat series images of Lake Gaoyou obtained are atmospheric-corrected surface reflectance data. The obtained image data are near-cloudless images (cloud cover <20%) during the submerged macrophyte (especially *P. crispus*) growth period in April and May.

This study employed a remote sensing-based automatic classification algorithm for the extraction of macrophytes, which is able to distinguish algal blooms, emergent/floating-leaved macrophytes, and submerged macrophytes in eutrophic lakes. The decision tree is composed of two vegetation indices and their respective thresholds (**Supplementary Figure 1**): the Aquatic Vegetation Index (AVI) (Luo et al., 2023) and the Normalized Difference Vegetation Index (NDVI). Here, AVI was calculated based on the humidity coefficient and reflectance of various bands after the Landsat tasseled cap transformation and was used to extract the macrophyte area (Equation 1). NDVI was used to extract the floating-leaved vegetation and emergent macrophyte (FEM) area (Equations 1, 2). The specific process is as follows: (1) By a threshold value *a*, the study area was divided into macrophyte areas and non-macrophyte areas: the pixel with AVI > *a* was identified as macrophyte and the remaining pixels were non-macrophyte. (2) In the macrophyte area, further classification was carried out by a threshold value *b*. The pixel with NDVI > *b* was classified as FEM and the remaining pixels were submerged macrophytes. Among them, the threshold *a* for AVI was a dynamic threshold, which varies for images acquired on different dates. It was obtained through a mixed linear model (Equation 3). The NDVI threshold was derived from extensive empirical data training, aimed at extracting FEM in Lake Gaoyou. The universal threshold *b* = 0.2 for NDVI was acquired through the threshold statistical graph obtained by the maximum gradient method. The AVI, NDVI, $S_{m,i}\left(\lambda \right)$ formula was expressed as Equations 1-3:


(1) $$\text{AVI}=-\sum_{i=1}^{6}k\left({\text{λ}}_{i}\right)R\left({\text{λ}}_{i}\right)$$

(2) $$NDVI=\left(R_{NIR}-R_{Red}\right)/\left(\;R_{NIR}+R_{Red}\right)$$

(3) $$S_{m,i}\left(\lambda \right)=p\times S_{W,i}\left(\lambda \right)+\left(1-p\right)\times S_{V}\left(\lambda \right)$$

where $k\left({\text{λ}}_{i}\right)$ represents the wetness coefficient of the tasseled cap transformation for band *i* in different satellite images. $R\left({\text{λ}}_{i}\right)$ represents the surface reflectance of the corresponding spectral band *i*; $R_{NIR}$ , $R_{Red}$ , and $R_{SWIR1}$ are the reflectances of the near-infrared, red, and short-wave infrared bands, respectively; $\lambda_{NIR}$ , $\lambda_{Red}$ , and $\lambda_{SWIR1}$ correspond to the central wavelengths of the near-infrared, red, and short-wave infrared bands, respectively; $S_{m,i}\left(\lambda \right)$ , $S_{W,i}\left(\lambda \right)$ , and $S_{V}\left(\lambda \right)$ represent the spectra of mixed materials, pure water, and pure vegetation, respectively; *p* represents the proportion of the pure water spectrum in the spectral mixture. Accuracy evaluation results of the classification confusion matrix are displayed in **Supplementary Table 1**.

Owing to the typical spectral characteristics of vegetation exhibited by FEM growing in the lakeshore zone, which has stronger spectral signals compared to submerged macrophytes, they are easily distinguishable from each other. *P. crispus* dominates the submerged macrophyte population in Lake Gaoyou, with the distribution of other species being quite limited (Tian et al., 2019; Xia et al., 2022). Additionally, considering that other submerged macrophytes are in their germination phase in April and May, while *P. crispus* is in its rapid growth phase with its stems closest to the water surface, exhibiting relatively strong spectral signals at this time. Therefore, we selected satellite images from April and May to identified the area of *P. crispus* in Gaoyou Lake. The remote sensing images from 2011 to 2013 and 2015 have high cloud content or contain stripes. By downloading Landsat 7 ETM and Landsat 8 OLI satellite data, visual interpretation was conducted to extract the area of *P. crispus* for these years.

### 2.3 Meteorological, hydrological, and water quality data

The meteorological factors, air temperature (Temp), wind speed (WS), and precipitation (PP) from 1984 to 2022, were obtained from the National Weather Science Data Center (https://data.cma.cn/). The hydrological factor [water level (WL)] data from 1984 to 2022 were obtained from the Jiangsu Provincial Hydrology and Water Resources Investigation Bureau. The water quality data from 2009 to 2022 were obtained from field surveys and sampling analysis.

The water quality survey conducted between 2009 and 2022 involved seven sampling sites (**Figure 1**), namely, two seasonal sampling sites across the lake (February, May, August, and November/December) and five monthly sampling sites. After the initial processing, the collected water samples were further detected and analyzed in the laboratory, ultimately yielding data on seven water quality parameters. Secchi depth (SD) was measured with the Secchi disk, and DO was measured with a portable multi-parameter water quality meter (YSI Professional Plus, USA). Surface, middle, and bottom water samples taken with a Plexiglas sampler were pooled and kept cool in a 1-L refrigerated container (at 4°C) and transported to the laboratory within 24 h. Total nitrogen (TN) was determined by potassium persulfate oxidation and UV spectrophotometry, and total phosphorus (TP) was determined by ammonium molybdate spectrophotometry. Ammonia nitrogen (NH_3_-N) was determined using the nano reagent photometric method, and the permanganate index (COD_Mn_) was determined using the dichromate method. Chlorophyll a (Chl-*a*) was determined using a spectrophotometer (UV-2450, Shimadzu Co., Ltd., Japan) after filtering known amounts of water through a GF/F (Whatman International Ltd., Maidstone, England) filter.

The flowchart of the grouping of environmental factors to analyze the area of *P. crispus* is shown in **Supplementary Figure 2**.

### 2.4 Data analysis

To understand the variation trend of *P. crispus* area, the “lm” function was used to analyze the long-term temporal changes of environmental factors in Lake Gaoyou. Subsequently, the “segmented” package was employed to fit segmented models to the time series of *P. crispus* area over the years. As *P. crispus* germinates in winter and blooms in spring, we examined the effects of environmental factors on the *P. crispus* area based on data of winter (from December of the previous year to March), spring (from April to May), and the entire year (from January to December). To identify the key environmental factors explaining the area of *P. crispus*, the “cor.test” function was employed to analyze the correlation between the area of *P. crispus* and meteorological factors, hydrological factors, and water quality factors. To perform stepwise regression analysis and select the main driving factors explaining the area of *P. crispus*, the “step” function was used. Subsequently, the “lm” function was employed to fit the area of *P. crispus* with the selected factors.

The “vegan” package was used for variation partitioning analysis to analyze the importance of two types of factors (hydrometeorological factors and water quality factors) in influencing the area of *P. crispus*.

The “ggplot2” package was used to visualize the above analysis results. In addition, *p <* 0.05 is considered to have a statistically significant difference in all analyses. Data analysis was performed using relevant packages in R version 4.3.2, and the plots were generated on both Origin and R platforms.

## 3 Results

### 3.1 Temporal changes in the environment factors

**Figure 2:**
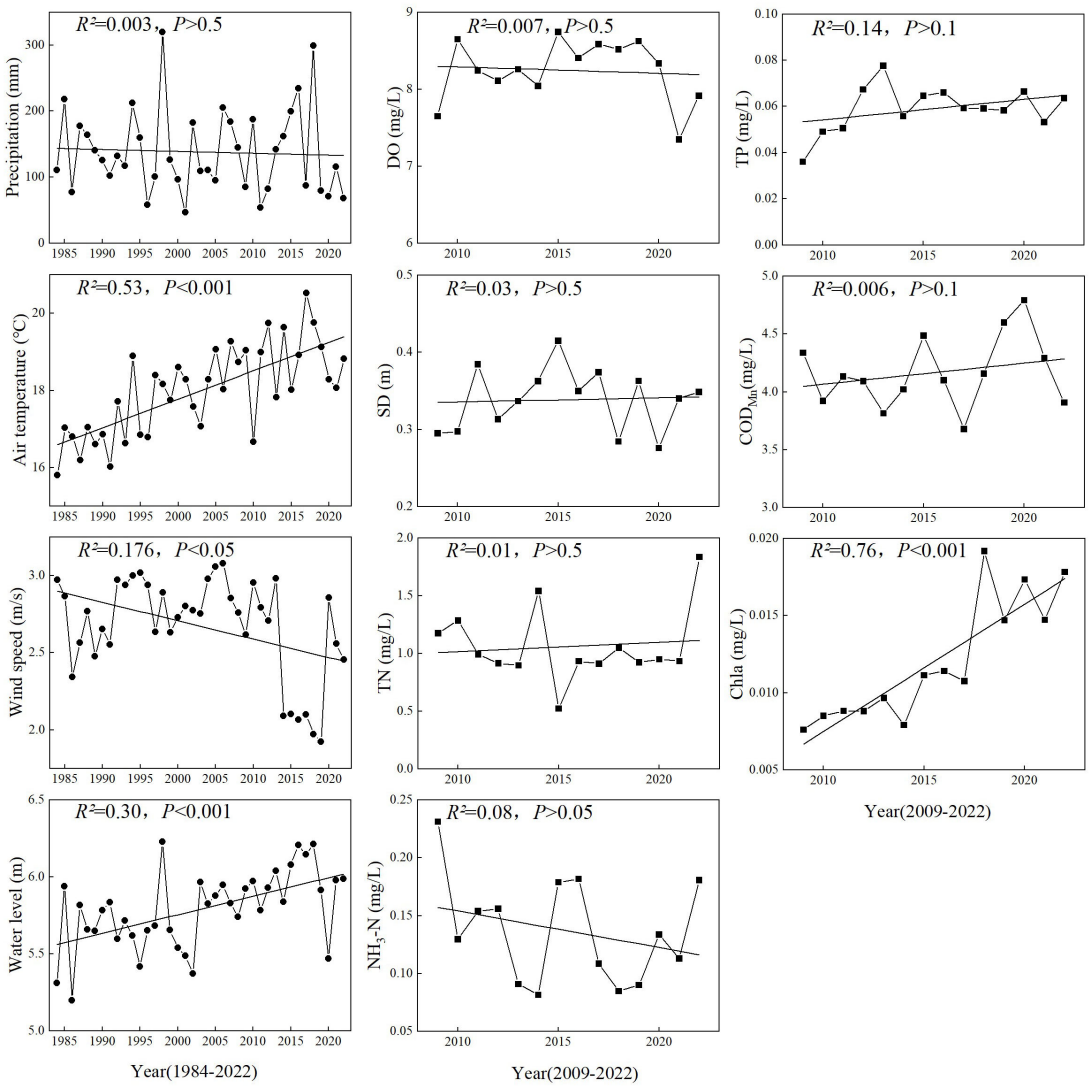
Temporal changes in hydrometeorological and water quality variables in spring. See the main text for abbreviations.

The hydrometeorological factors during the bloom period of *P. crispus* showed significant changes from 1984 to 2022 (**Figure 2**, **Supplementary Figures 3**, **4**). For example, in spring, the Temp (18.22 ± 1.08°C) increased markedly over time and reached its maximum of 20.53°C in 2017, followed by a decreasing trend between 2017 and 2020. Conversely, WS (2.65 ± 0.35 m/s) declined significantly from 2013 to 2019 and reached its minimum of 1.92 m/s, with a noticeable rebound from 2019 to 2020, followed by a subsequent decline. The annual average WL (5.79 ± 0.25 m) increased significantly over time and reached its maximum of 6.21 m in 2018. From 2009 to 2022, the main water quality factors of Lake Gaoyou also showed significant temporal variations. For example, in spring, Chl-*a* (0.012 ± 0.004 mg/L) increased markedly and reached its maximum of 0.019 mg/L in 2018. DO (8.25 ± 0.39 mg/L) and SD (0.34 ± 0.04 m) showed a fluctuating trend. TP (0.06 ± 0.01 mg/L) increased from 2009 to 2013 and peaked at 0.08 mg/L in 2013, followed by fluctuating changes. TN (1.06 ± 0.31 mg/L) showed considerable fluctuation, mostly remaining at approximately 1.0 mg/L. COD_Mn_ (4.17 ± 0.30 mg/L) showed an overall slight increasing trend and reached its maximum of 4.79 mg/L in 2020, followed by a declining trend.

**Figure 3:**
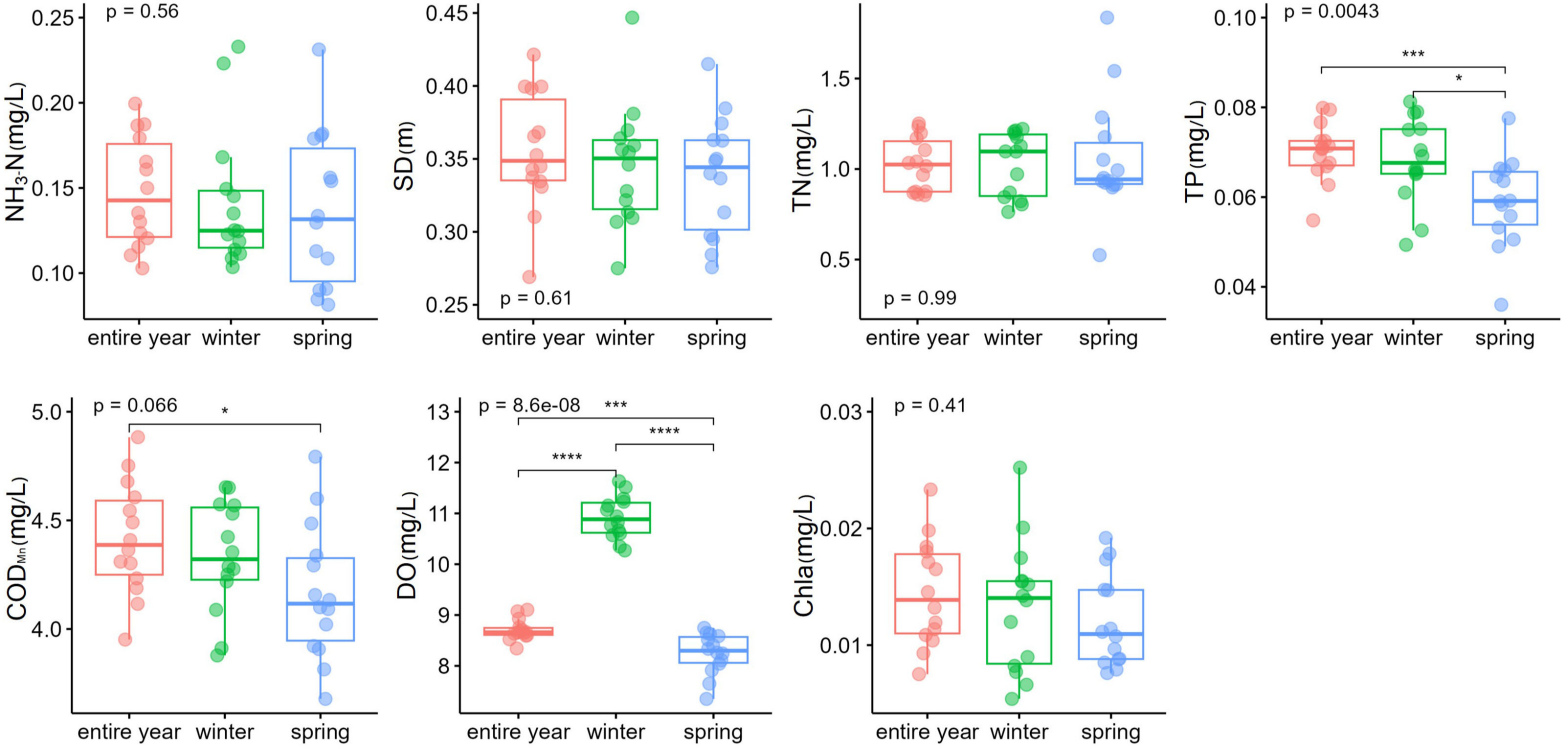
Comparisons of water quality in different seasons. *p < 0.05; ***p < 0.001. ****p < 0.0001..

In addition, some water quality factors showed a significantly seasonal difference (**Figure 3**). The investigated lake in spring had a median TP concentration of 0.059 (0.036–0.076) mg/L and a median COD_Mn_ concentration of 4.17 (3.68–4.79) mg/L. However, TP and COD_Mn_ concentration for the entire year were 0.070 (0.054–0.080) mg/L and 4.41 (3.95–4.88) mg/L, respectively. TP and COD_Mn_ in spring were significantly lower than the concentration for the entire year (*p <* 0.001 for TP and *p <* 0.05 for COD_Mn_). DO also showed pronounced differences among three periods and had a significantly higher median concentration of 10.92 (10.27–11.63) mg/L in winter than the other two (*p <* 0.001).

### 3.2 Long-term trends and the spatial distribution of *P. crispus*

**Figure 4:**
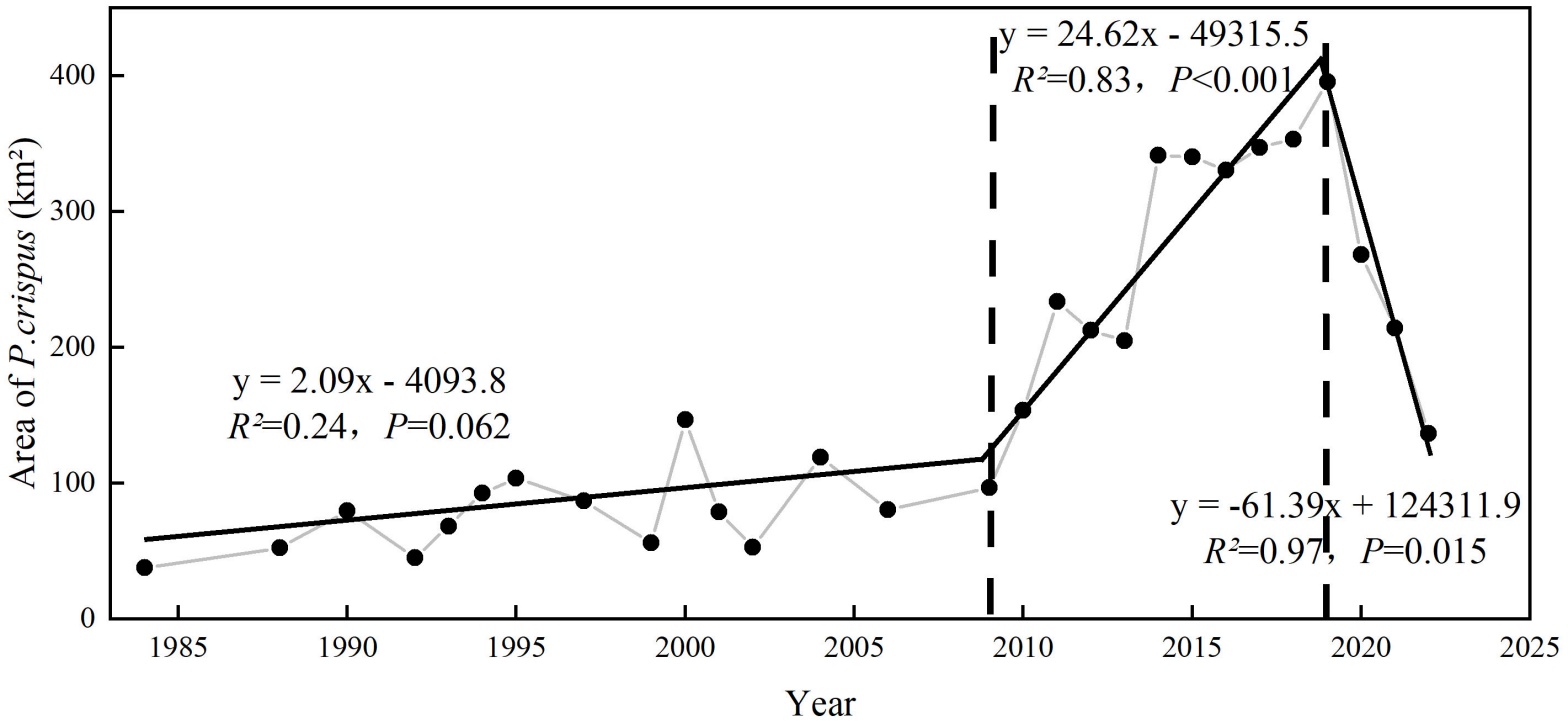
The variation in the area of *P. crispus* in Lake Gaoyou from 1984 to 2022.

The segmented linear fitting results indicated that the variation in the area of *P. crispus* between 1984 and 2022 can be divided into three time periods (**Figure 4**). From 1984 to 2009, the area of *P. crispus* in Lake Gaoyou slightly increased from approximately 37.86 km² to approximately 100 km², with a relatively low distribution area. From 2010 to 2019, the area of *P. crispus* began to markedly increase (*p <* 0.001), exhibiting a large-scale bloom trend with an average area of 291.31 km², and reached its maximum of 395.51 km². After 2019, the area of *P. crispus* showed a rapid decrease (*p <* 0.05) and reached 136.78 km² in 2022.

**Figure 5:**
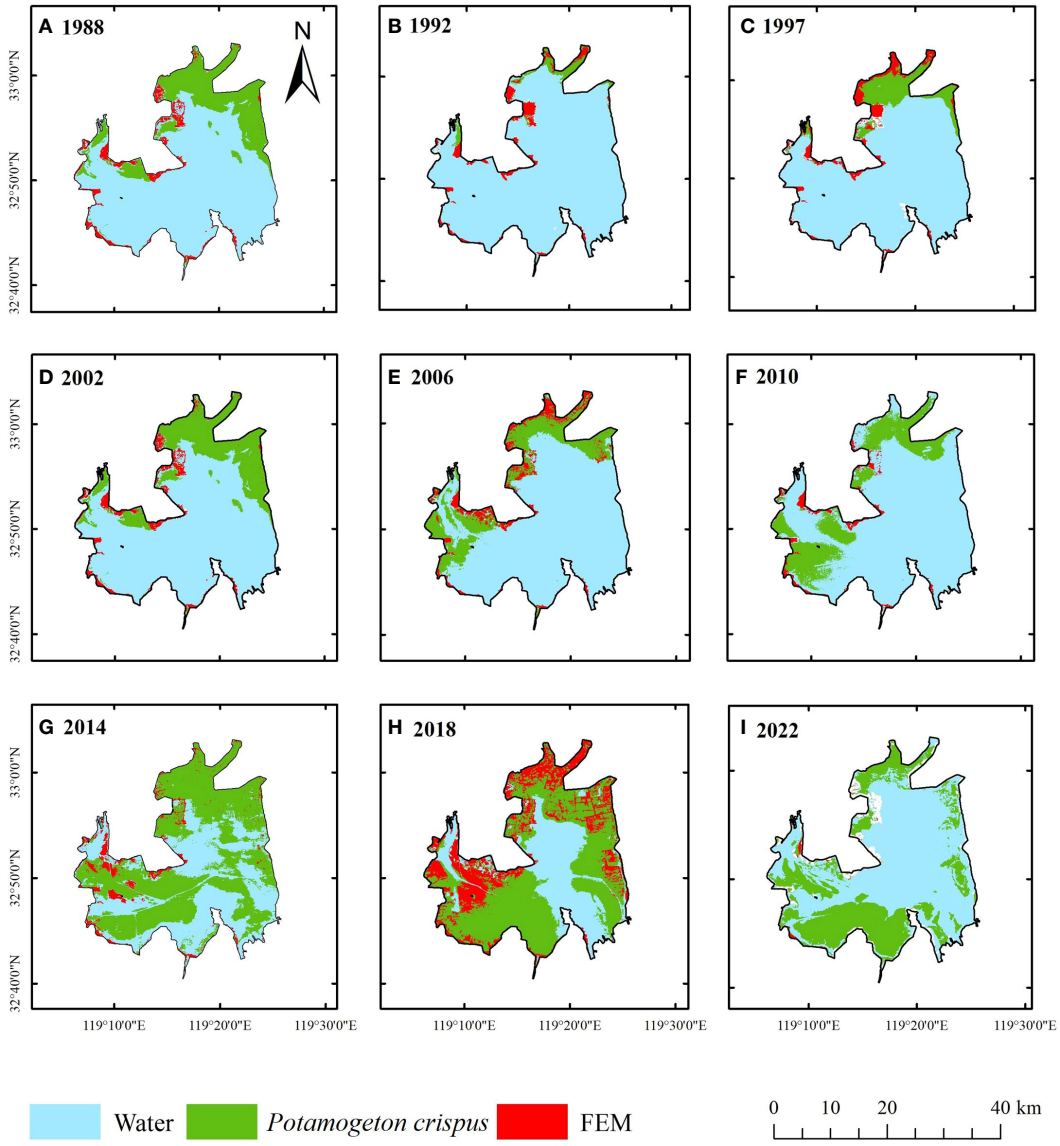
Changes in the spatial distribution of the *P. crispus* area in Lake Gaoyou from 1984 to 2022. **(A)** 1988 **(B)** 1992 **(C)** 1997 **(D)** 2002 **(E)** 2006 **(F)** 2010 **(G)** 2014 **(H)** 2018 **(I)** 2022. FEM, floating-leaved and emergent macrophyte.

From a spatial perspective, *P. crispus* was primarily distributed in the western and northern parts of Lake Gaoyou, with less distribution in the central and southeastern parts of the lake (**Figure 5**; **Supplementary Figure 5**). From 1984 to 2010, *P. crispus* in Lake Gaoyou transitioned from a scattered distribution in the northern part of the lake to extending towards the western part, gradually increasing in area. From 2010 to 2019, *P. crispus* continued to extend towards the southeastern part of the lake and spread from the surrounding areas towards the central part of the lake, rapidly expanding in area. From 2019 to 2022, the area covered by *P. crispus* decreased rapidly as it receded from the central part of the lake towards the surrounding areas.

### 3.3 Factors driving the distribution pattern succession of *P. crispus*

#### 3.3.1 Correlation analysis

**Figure 6:**
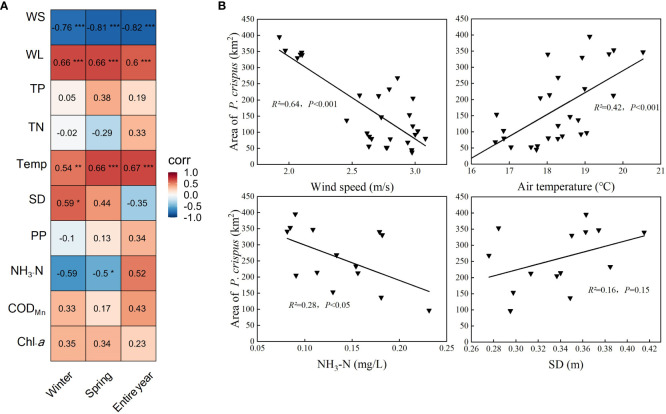
Correlation analysis between the area of *P. crispus* and environmental factors. **(A)** Relationships between the area of *P. crispus* and environmental factors. The numbers in each grid represent the correlation coefficients between the area of *P. crispus* and environmental factors. **(B)** Linear fitting of the area of *P. crispus* and environmental factors in spring. * *p* < 0.05; ** *p* < 0.01; *** *p* < 0.001.

The results of the Pearson correlation analysis showed that the area of *P. crispus* in Lake Gaoyou showed a significantly positive correlation with Temp and WL (*p <* 0.001) and a significantly negative correlation with WS (*p <* 0.001). The correlations between water quality factors for different periods and the area of *P. crispus* varied (**Figure 6A**): In the analysis of environmental factors in winter, the area of *P. crispus* was positively correlated with SD (*p <* 0.05). In the analysis of environmental factors in spring, the area of *P. crispus* was negatively correlated with NH_3_-N (*p <* 0.05).

#### 3.3.2 Stepwise regression analysis

The stepwise regression analysis showed that the five most important factors influencing the change in *P. crispus* area in Lake Gaoyou were WS, Temp, WL, NH_3_-N, and SD (**Table 1**). In particular, the area of *P. crispus* was found to correlate closely with hydrometeorology, and the contribution of WS was particularly significant (*p <* 0.001). The area of *P. crispus* consistently showed a negative association with WS and a positive relationship with SD in the analysis of three periods (*p <* 0.05). In the analysis of entire-year and winter environmental factors, Temp and WL (*p <* 0.05) were two important factors, both of which were positively correlated with the area of *P. crispus* (**Supplementary Figures 7**, **8**). In the analysis of entire-year and spring environmental factors, the area of *P. crispus* showed a significantly negative correlation with NH_3_-N (*p <* 0.05) (**Figure 6B**), and the relative contributions of the variables to explain the total variation were NH_3_-N > SD. The regression models selected with significance (*p <* 0.001) between hydrometeorology and the area of *P. crispus* for different periods had a total explanation of 72.6%, 75.2%, and 64.0%, respectively. The regression models selected with significance between water quality and the area of *P. crispus* for different periods had a total explanation of 36.4%, 34.0% (*p <* 0.05), and 44.9% (*p <* 0.05), respectively.

**Table 1:** The multiple regression model results for the area of *P. crispus* and environmental factors.

Area	Factor	*R*² (%)	*F*	*p*	Variables	Standardized coefficients
Entire year	Hydrometeorology	72.6	36.75	<0.001	WS	−0.674 ***
					Temp	0.290 *
	Water quality	36.4	3.146	0.083	NH_3_-N	−0.452
					SD	0.404
Winter	Hydrometeorology	75.2	37.86	<0.001	WS	−0.837***
					WL	0.288*
	Water quality	34.0	6.167	<0.05	SD	0.583*
Spring	Hydrometeorology	64.0	46.12	<0.001	WS	−0.800 ***
	Water quality	45.0	4.489	<0.05	NH_3_-N	−0.537*
					SD	0.407

*p < 0.05; ***p < 0.001.

#### 3.3.3 Variation partitioning analysis

**Figure 7:**
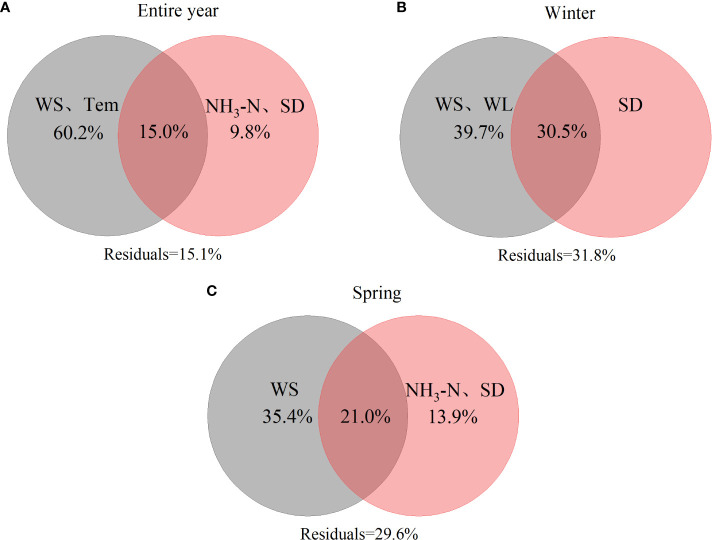
The differences in the relative contributions of hydrometeorology and water quality to the area of *P. crispus*. **(A)** entire year, **(B)** winter, **(C)** spring. Values < 0 are not shown.

The results of the variation partitioning analysis showed that the *P. crispus* presented significant differences among the different periods. In the analysis of entire environmental factors, WS and Temp accounted for 60.2% of the variation in the *P. crispus* area, with their interaction with NH_3_-N and SD explained 15.0% (**Figure 7A**). For winter environmental factors, WS and WL explained 39.7% of the variation in the *P. crispus* area, with the shared fraction with SD explained 30.5% (**Figure 7B**). For spring environmental factors, WS explained 35.4% of the variation in the *P. crispus* area, with their interaction with NH_3_-N and SD explained 21.0% (**Figure 7C**).

The variation partitioning analysis for all three periods indicated that hydrometeorological factors (Temp, WS, and WL) accounted for the high amount of variation in the *P. crispus* area in Lake Gaoyou compared to water quality (NH_3_-N, and SD), playing a dominant role in driving the changes in the area of *P. crispus*. Additionally, the interaction between hydrometeorology and water quality explained a higher proportion of variation in the area of *P. crispus* in winter, whereas its amount of explanation was lower for the entire year.

## 4 Discussion

### 4.1 Main driving factors affecting the distribution of *P. crispus*

The five most important factors influencing the change in the *P. crispus* area in Lake Gaoyou were WS, Temp, WL, NH_3_-N, and SD. Among them, WS played a crucial role in the variation of the *P. crispus* area (**Table 1**). On one hand, WS can affect the growth of *P. crispus* by influencing the hydrodynamic conditions of the lake. Macrophytes in lakes are often affected by water flow resistance, which is frequently over 25 times more resistant compared to terrestrial vegetation under similar wind speeds (Denny and Gaylord, 2002). Submerged macrophytes will also show different biomechanical characteristics subject to the wind and wave conditions (Zhu et al., 2015). Consequently, most submerged macrophytes float to the water’s surface when strong winds and waves cause stems to break or entire plants to be uprooted. This causes a significant decrease in submerged macrophytes in shallow lakes, or even their disappearance, which reduces the macrophytes’ biomass and coverage (Riis and Biggs, 2003; Yang et al., 2004; Breugnot et al., 2008; Angradi et al., 2013). Consistent with previous findings, in this study, WS was an important variable that negatively contributed to the *P. crispus* area (**Table 1**); the *P. crispus* area showed an increasing trend with declining WS from 2010 to 2019. On the other hand, wind speed is often significantly negatively correlated with transparency in shallow lakes (Soria et al., 2021), which is similar to the results of this study (**Supplementary Figure 9**). Increased wind speeds can intensify hydrodynamic disturbances in lakes, causing lake sediments to appear in resuspension and reducing transparency (Soria et al., 2021). A decline in transparency inhibits plant photosynthesis, which was unfavorable for the growth of *P. crispus* in Lake Gaoyou. According to the spatial distribution of *P. crispus* in Lake Gaoyou, the area of *P. crispus* in the southeastern part of the lake was perennially small. This may be due to the fact that this area serves as a flood channel where the southeast monsoon prevails, which was prone to stronger hydrodynamic disturbances leading to lower water transparency, thus inhibiting the growth of *P. crispus*.

Generally, water depth has been considered a significant factor influencing the growth of submerged macrophytes. Typically, there is a negative correlation between water depth and the biomass of submerged macrophytes (Li et al., 2021). However, in shallow lakes, increasing the water depth probably creates a favorable growing habitat for submerged macrophytes by changing the temperature, DO content, and underwater light intensity and by expanding the growth space for these plants (Fu et al., 2014, 2018; Su et al., 2018). On the other hand, changes in water depth or level often interact with factors such as wind speed to influence submerged macrophytes (Van Zuidam and Peeters, 2015). An increase in water depth often leads to a decrease in the intensity of wind–wave disturbances (Søndergaard et al., 2003). This, to some extent, mitigated the negative effects of wave disturbance on SD, thereby promoting the expansion of the *P. crispus* area.

The stepwise linear regression model conducted in our study indicated that NH_3_-N concentration and SD were the two important water quality variables that contributed to the area of *P. crispus* (**Table 1**). The improvement in water transparency helps to increase the underwater light intensity, thereby improving photosynthesis efficiency in plants and consequently promoting the growth of *P. crispus* (Wang et al., 2017). Previous studies also concluded that macrophytes showed a decreasing trend due to a deteriorating underwater light environment. In our study, NH_3_-N negatively contributed to the area of *P. crispus.* High concentrations of ammonium ions can interfere with the nitrogen metabolism of *P. crispus* (Zhang YL et al., 2016; Dong et al., 2022), and physiological stress caused by high concentrations of ammonia nitrogen in the water can also lead to a decrease in the biomass of *P. crispus* (Cao et al., 2015). Several laboratory and field studies have demonstrated that macrophytes in lakes can experience damage and even disappear when there is a high concentration of nitrogen, particularly ammonia nitrogen (Moss et al., 2012; Olsen et al., 2015; Yu et al., 2015; Zhang YL et al., 2016). The conclusion has also been confirmed by site-specific observation and remote sensing mapping (Zhang YL et al., 2016). These findings imply that the increasing SD and decreasing NH_3_-N concentration in Lake Gaoyou may enhance the area of *P. crispus* from 2010 to 2019.

Additionally, *P. crispus* has an interactive relationship with water quality. Submerged macrophytes improve local water transparency by reducing sediment resuspension and absorbing nutrients from the water; thus, there is a positive feedback relationship between submerged macrophyte and local water transparency in shallow lake ecosystems (Su et al., 2019). Similarly, *P. crispus* needs to absorb nutrients such as ammonia nitrogen from sediment and water through its roots and stems to support growth (Li et al., 2015).

### 4.2 Seasonal characteristics of driving factors

The influence of environmental factors in different seasons on the area of *P. crispus* in Lake Gaoyou varied (**Table 1**). In this study, NH_3_-N significantly contributed to the fluctuation of *P. crispus* area in spring, while no statistical difference was observed for NH_3_-N in winter. Conversely, WL in winter significantly contributed to the variation in the *P. crispus* area, while no statistical difference was observed for water level in spring.

Water level itself generally does not directly affect macrophytes but often influences plant growth by altering other environmental factors (Van Zuidam and Peeters, 2015). Increasing fluctuation frequency increases disturbance to plants, leading to increased nutrient loss and tissue damage (Bornette et al., 2008). High-frequency fluctuations in the water level can also lead to the resuspension of sediments, increasing water turbidity, thereby inhibiting the growth of macrophytes (Coops and Hosper, 2002; Luo et al., 2015). In May, Lake Gaoyou experiences a period of rising water levels, with significant fluctuations in water levels (Jiang et al., 2023). The flow velocity may rise simultaneously with water level during this period (Zhang et al., 2017). The rising flow velocity intensifies water disturbance, which is unfavorable for the growth of *P. crispus*. Compared to spring, water level fluctuations were smaller in winter, which may be the reason why the positive effect of WL changes on the expansion of the *P. crispus* area was more significant in winter.

The interactions between NH_3_-N and *P. crispus* showed a seasonal difference. On one hand, in a shallow eutrophic water body, macrophytes can assimilate a large amount of nitrogen from sediments via their roots during the growing season (Xie et al., 2013). The efficiency of assimilating and utilizing nitrogen increases when *P. crispus* blooms in spring. On the other hand, toxic effects cause the various growth indices of *P. crispus*, such as leaf length, leaf mass, and root length, to decline with increasing nitrogen and ammonia concentrations (Yu et al., 2015). Therefore, in Lake Gaoyou, the relatively higher concentration of NH_3_-N in spring compared to winter may explain a more noticeable inhibitory effect of NH_3_-N on the growth of *P. crispus* during spring.

The temperature was only screened as a significant influencing factor in the factor analysis at the annual scale. This was primarily because the multivariate stepwise regression algorithm aimed to balance model stability and simplicity. Because of the significant correlation between factors (such as Temp and WS, *p <* 0.05), where WS coincided with Temp covariation, there was some redundancy in information (**Supplementary Figure 6**). Once WS entered the model, Temp could not enter to the same extent. However, from the correlation coefficients, it could be observed that Temp was significantly correlated with the area of *P. crispus*. Research related to the influence of temperature on *P. crispus* indicated that warming treatments significantly increased plant height and total biomass (Yan et al., 2021). As a submerged macrophyte that grows in winter and spring, *P. crispus* will enter the growing season earlier and occupy more spatial ecological niches as the winter gets warmer (Li et al., 2018). Consistent with previous studies, the results of this study demonstrate that an increase in Temp had a positive effect on the growth of *P. crispus* in Lake Gaoyou (**Table 1**; **Figures 2**, **4**).

### 4.3 Relative importance of hydrometeorology and water quality

The primary influencing factors for the growth of *P. crispus* in Lake Gaoyou were hydrometeorological factors (Temp, WS, and WL), which fits with our hypothesis. Moreover, the relative importance of the interaction between hydrometeorology and water quality varied across different periods. The interaction between hydrometeorology and water quality showed a higher explanation in winter, whereas it was lower over the entire year (**Figure 7**).

Against the backdrop of global climate change, increasing water temperatures, storm events, and associated long-term flooding have had a significant impact on the health of aquatic ecosystems dominated by macrophytes worldwide, thereby affecting the growth of macrophytes in lakes (Zhang et al., 2017). Research on large-scale vegetation in global lakes indicated that climate variables have a greater impact on species selection at a large spatial scale (García-Girón et al., 2020). Similar to previous studies, at the large spatial scale of Lake Gaoyou, climate had a more significant impact on the growth of *P. crispus* compared to anthropogenic activities. Furthermore, temperature changes have a significant impact on the growth of submerged macrophytes. For example, *Elodea canadensis* biomass increases directly with warmer temperatures as opposed to nutrient enrichment (Wu et al., 2021). Similarly, the drastic fluctuations in Temp in the Lake Gaoyou area also have significantly influenced the changes in the area covered by *P. crispus*.

Climate change is not a uniform warming process; its impact on winter is particularly noticeable (Franssen and Scherrer, 2008). Macrophytes can overwinter in an aboveground form under warmer winter (Havens et al., 2015). Warmer winters increase the number of branch and total biomass of macrophytes, thereby enhancing overwinter survival rates (Liu et al., 2016). Additionally, this study revealed that the interaction between winter WS and SD was more significant. It was possible that winter WS affected the growth of *P. crispus* via influencing SD, making the impact of winter WS on the expansion of *P. crispus* more significant. Additionally, this study revealed that the interaction between winter WS and SD was more significant (*p <* 0.05) (**Supplementary Figure 9**). It was possible that winter WS affected the growth of *P. crispus* by influencing SD, making the impact of winter WS on the expansion of *P. crispus* more significant.

## 5 Conclusion

Ensuring adequate appropriate coverage of submerged macrophyte growth is vital for maintaining water quality and ecosystem stability. The area of *P. crispus* in Lake Gaoyou showed a slight increase from 1984 to 2009, followed by a marked increase from 2010 to 2019, and then a decline after 2020. We found that the variation in the *P. crispus* area was highly influenced by WS, Temp, WL, NH_3_-N, and SD in Lake Gaoyou and showed seasonality in response to hydrometeorology and water quality parameters. Hydrometeorology factors appeared to exert a more substantial influence on the area covered by *P. crispus* than water quality parameters. The significantly decreasing WS and the increasing Temp and WL resulted in explosive trends in the area of *P. crispus*. Overall, our study revealed the long-term distribution pattern of *P. crispus* in Lake Gaoyou and identified key factors regulating the distribution of *P. crispus*. The proliferation of specific species would disrupt water quality and aquatic ecosystem stability. Therefore, effective lake management should include enhanced macrophyte monitoring and timely intervention measures to counteract the excessive growth of specific species, thereby safeguarding water resources for the China ER-SNWDP.

## Data availability statement

The raw data supporting the conclusions of this article will be made available by the authors, without undue reservation.

## Author contributions

KY: Writing – original draft, Writing – review & editing, Formal analysis, Visualization, Methodology. YY: Writing – original draft, Writing – review & editing, Data curation. YX: Writing – review & editing, Data curation. SW: Writing – review & editing, Data curation. MG: Writing – original draft, Data curation, Investigation. KP: Writing – review & editing, Data curation, Methodology. JL: Writing – review & editing, Methodology, Visualization. JG: Writing – review & editing. YC: Conceptualization, Formal analysis, Funding acquisition, Methodology, Writing – original draft, Writing – review & editing.
