# Supplementary material for: Hydrometeorological conditions drive long-term changes in the spatial distribution of Potamogeton crispus in a subtropical lake
Source: Front Plant Sci. 2024 Jul 9;15:1424300. doi: 10.3389/fpls.2024.1424300 (PMC11263109; doi:10.3389/fpls.2024.1424300)
Supplement: Supplementary file 1 [file DataSheet_1.docx]

**Figures**


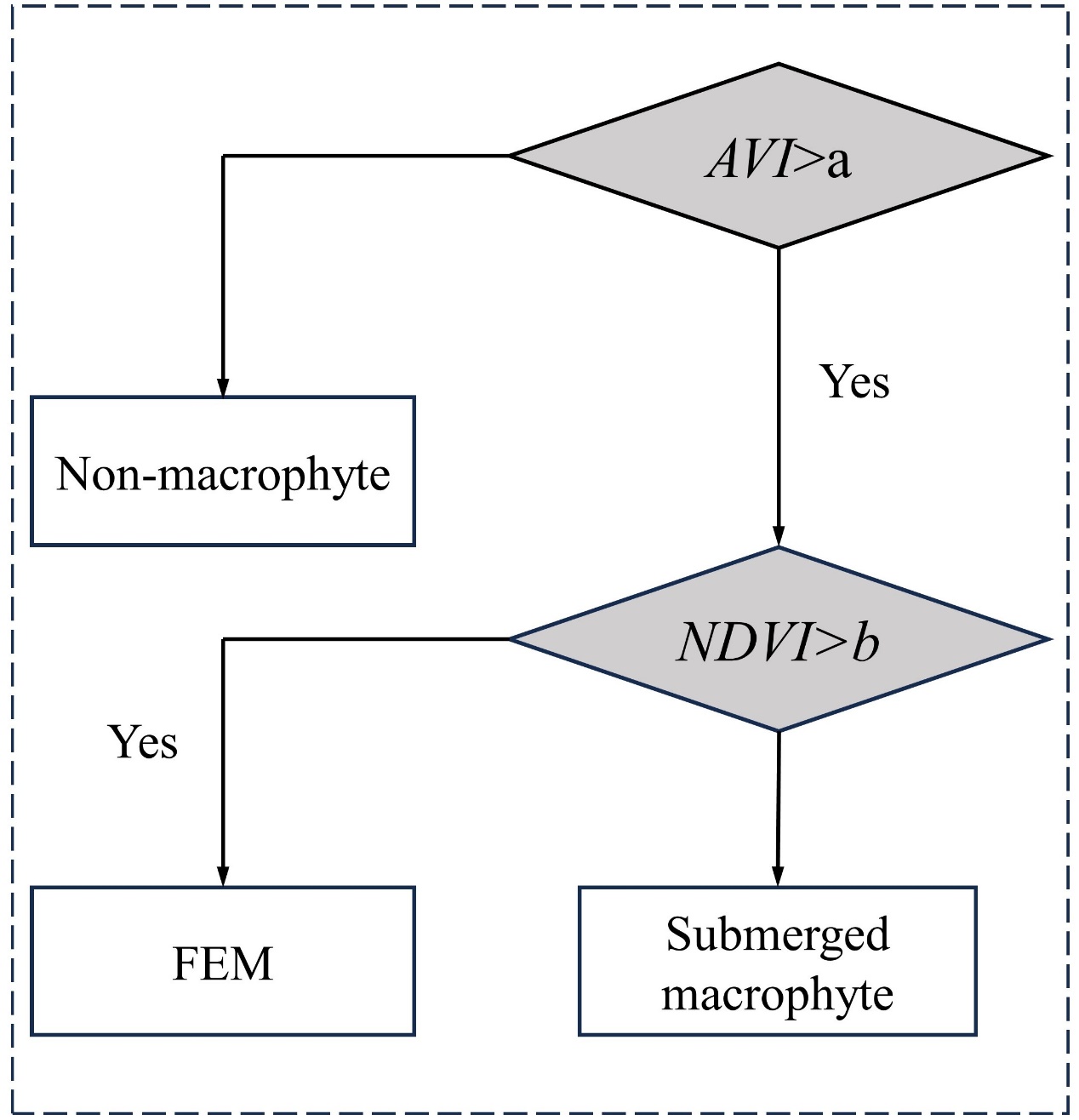


**Supplementary Figure 1.** Classification decision tree for submerged macrophyte and FEM. FEM, floating-leaved and emergent macrophyte.


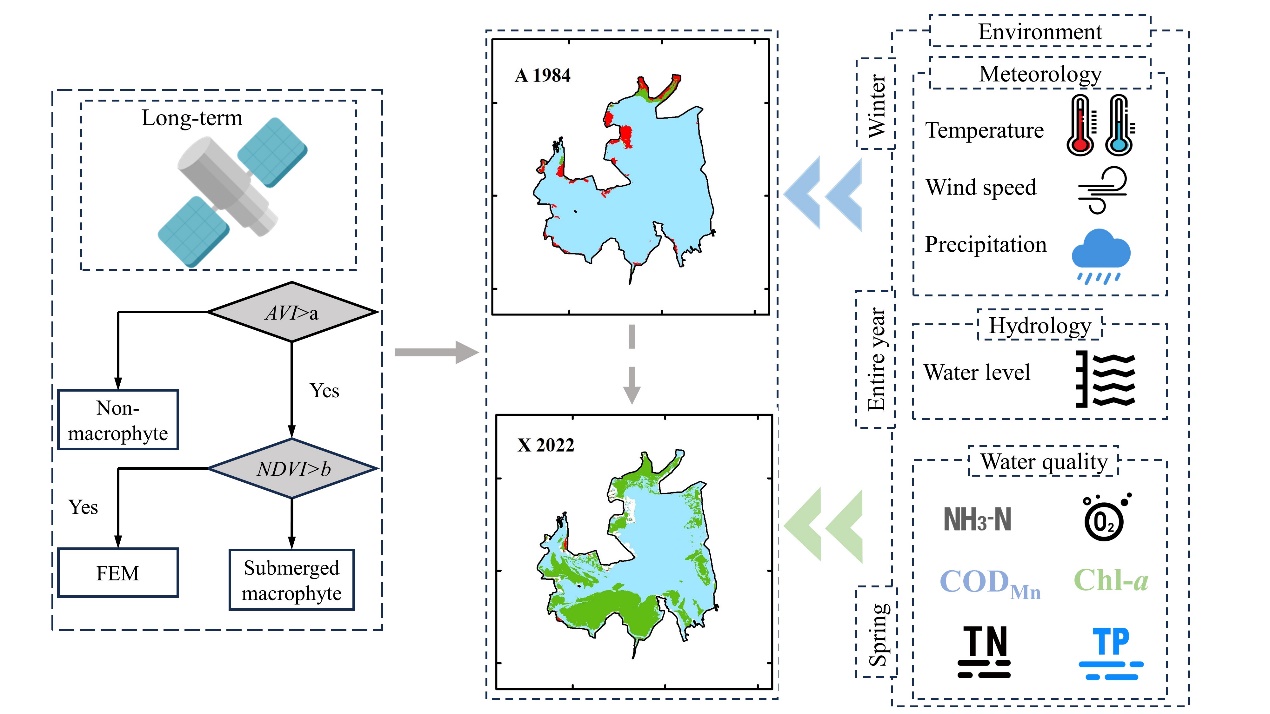


**Supplementary Figure 2.** Flowchart of the process of remote sensing data analysis and environmental factors analysis related to the area of *P. crispus*


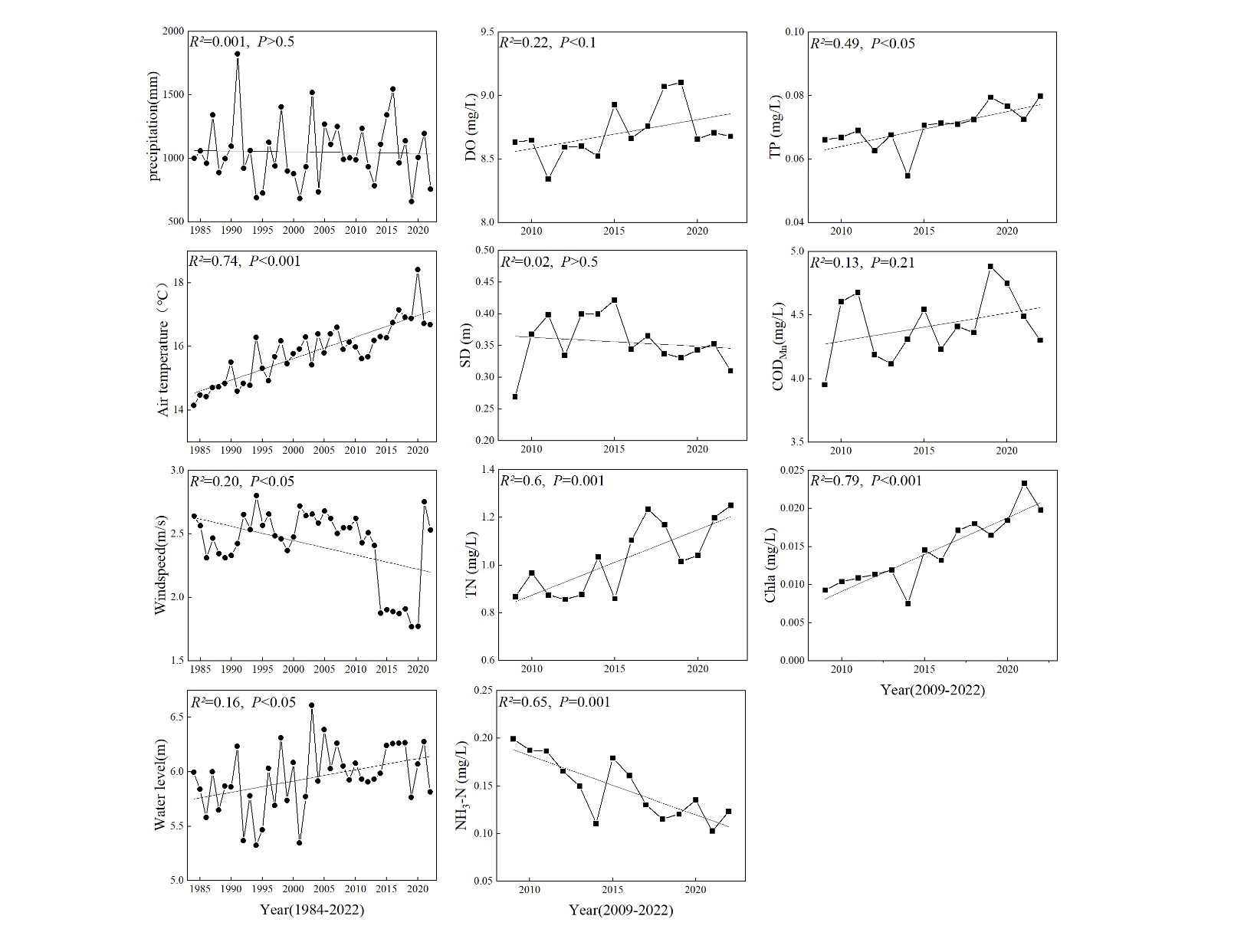


**Supplementary Figure 3.** Temporal changes in hydrometeorological and water quality variables in entire-year. Abbreviations see the main text.


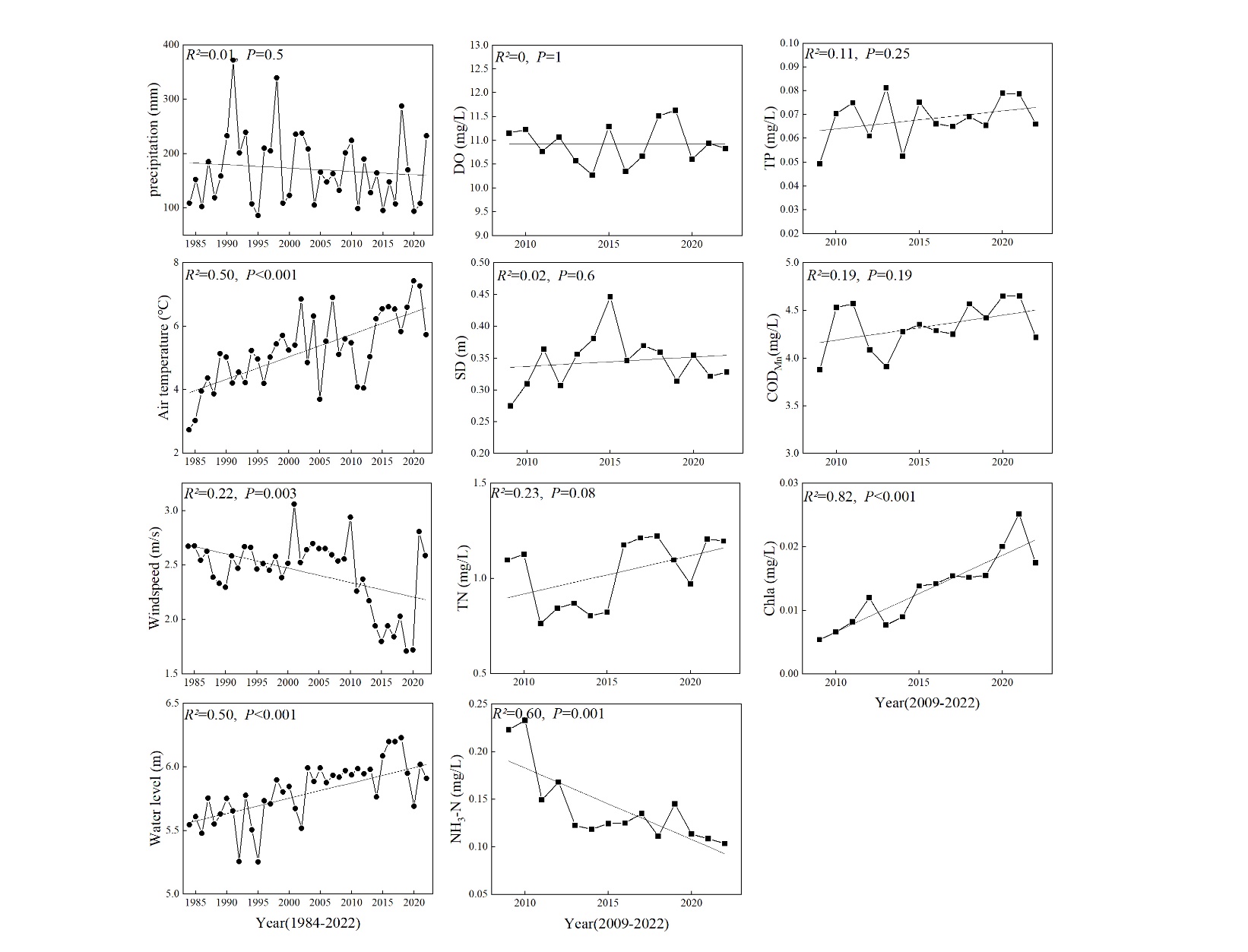


**Supplementary Figure 4.** Temporal changes in hydrometeorological and water quality variables in winter. Abbreviations see the main text.
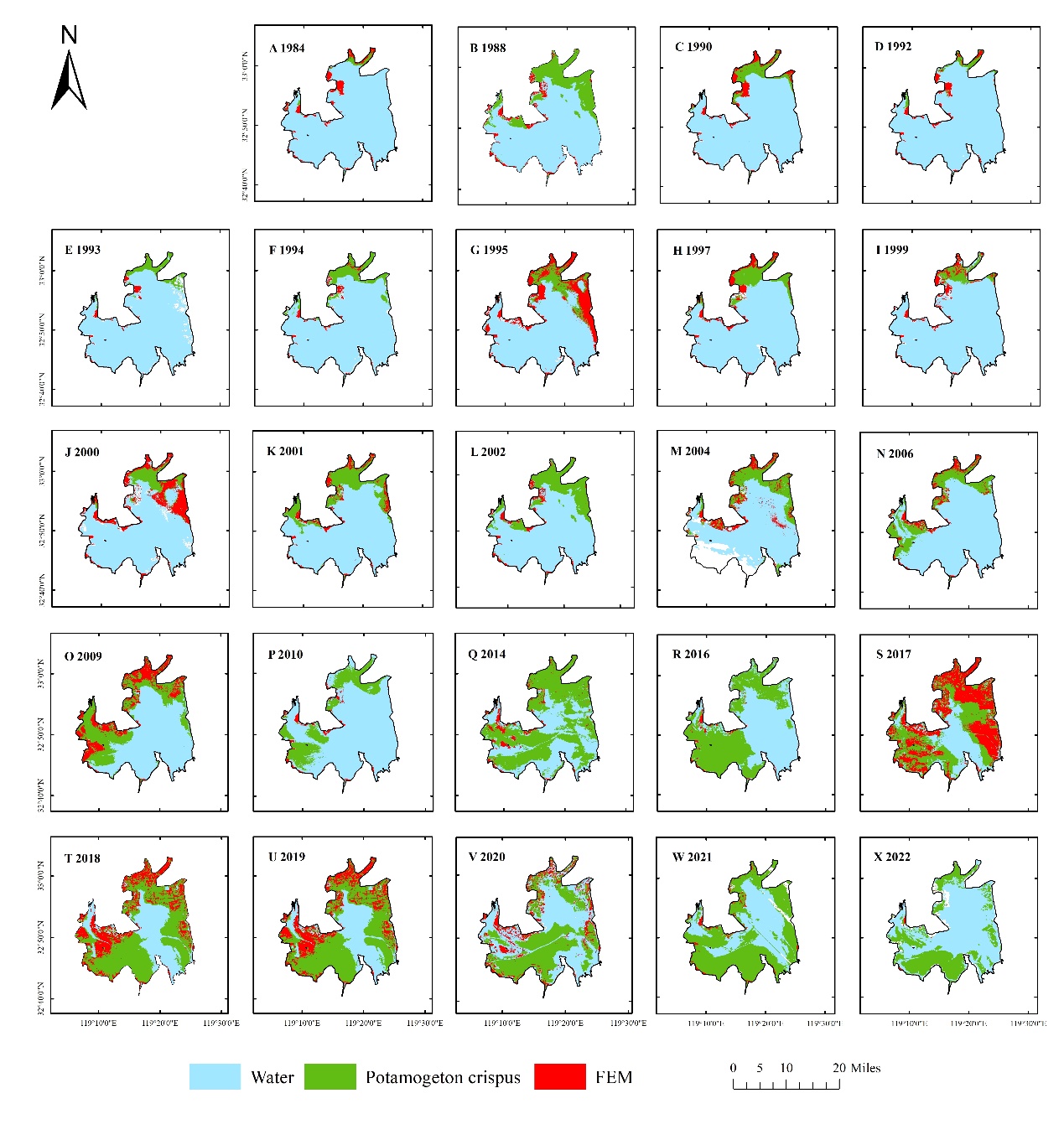


**Supplementary Figure 5.** Changes in spatial distribution of *P. crispus* area in Lake Gaoyou from 1984 to 2022. FEM, floating-leaved and emergent macrophyte.


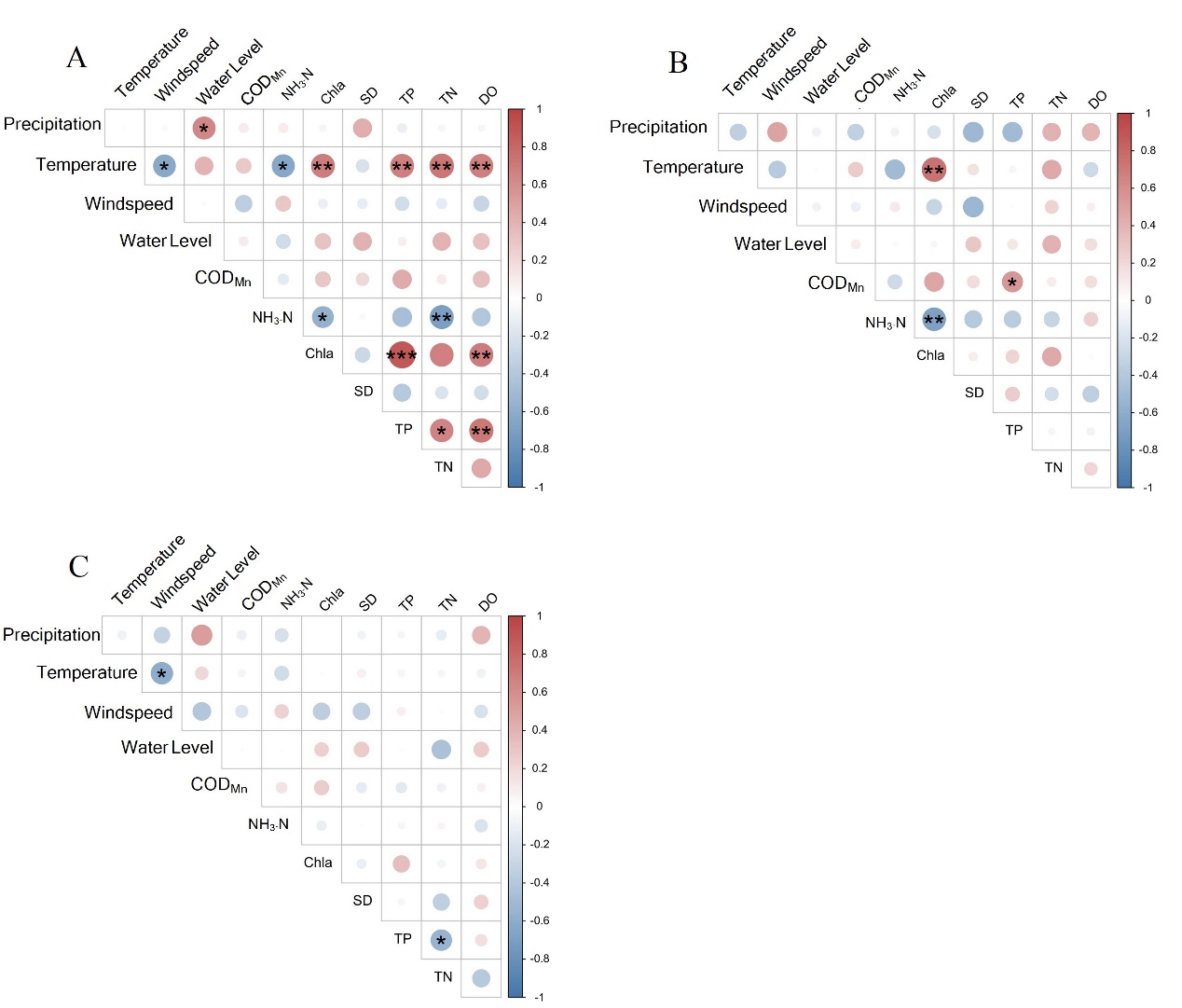


**Supplementary Figure 6.** Correlation Analysis between environmental factors in (A) entire year, (B) winter and (C) spring. **P* < 0.05；** *P* < 0.01；*** *P* < 0.001.

~~
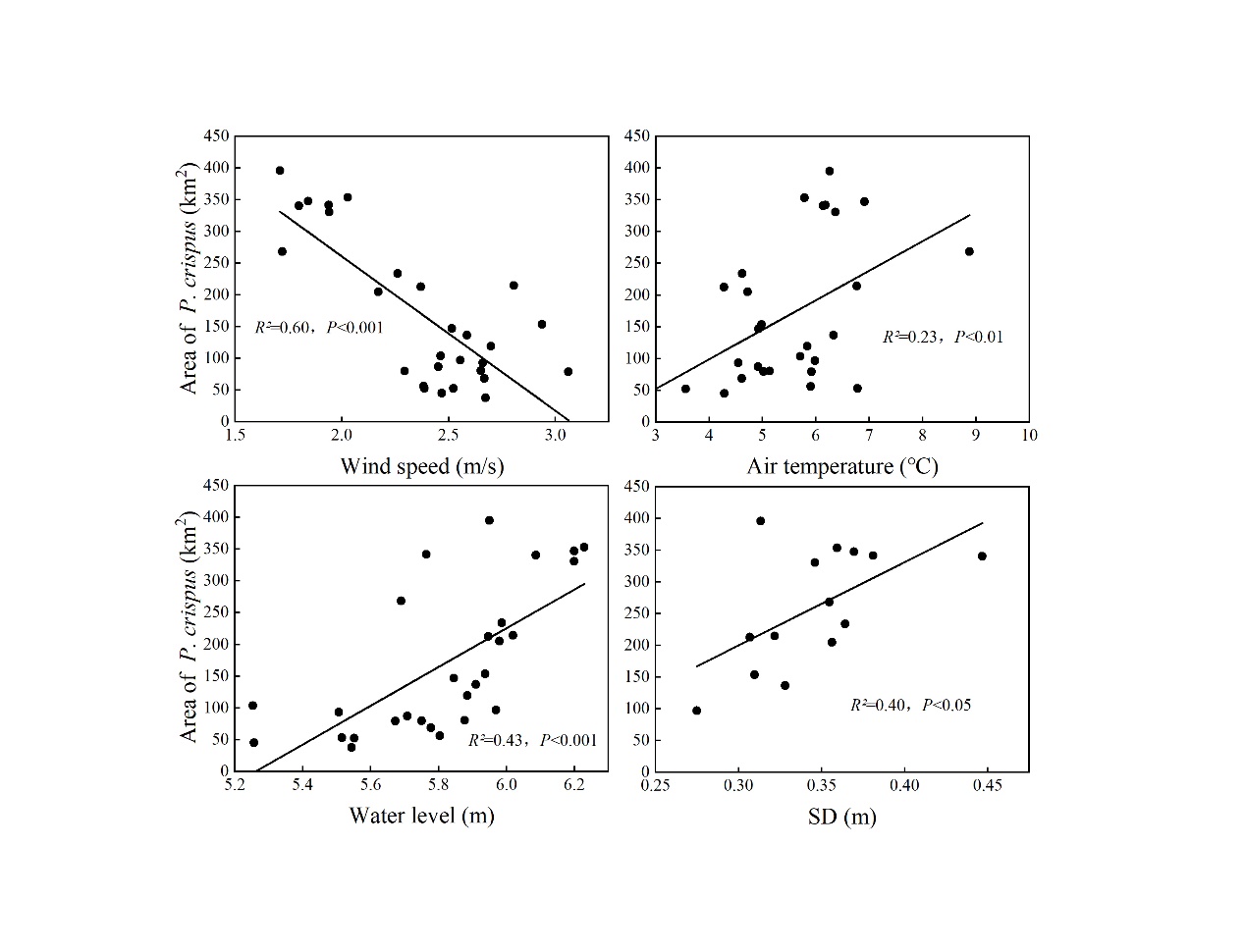
~~

**Supplementary Figure 7.** Linear fitting of the area of *P. crispus* and environmental factors in winter.

~~
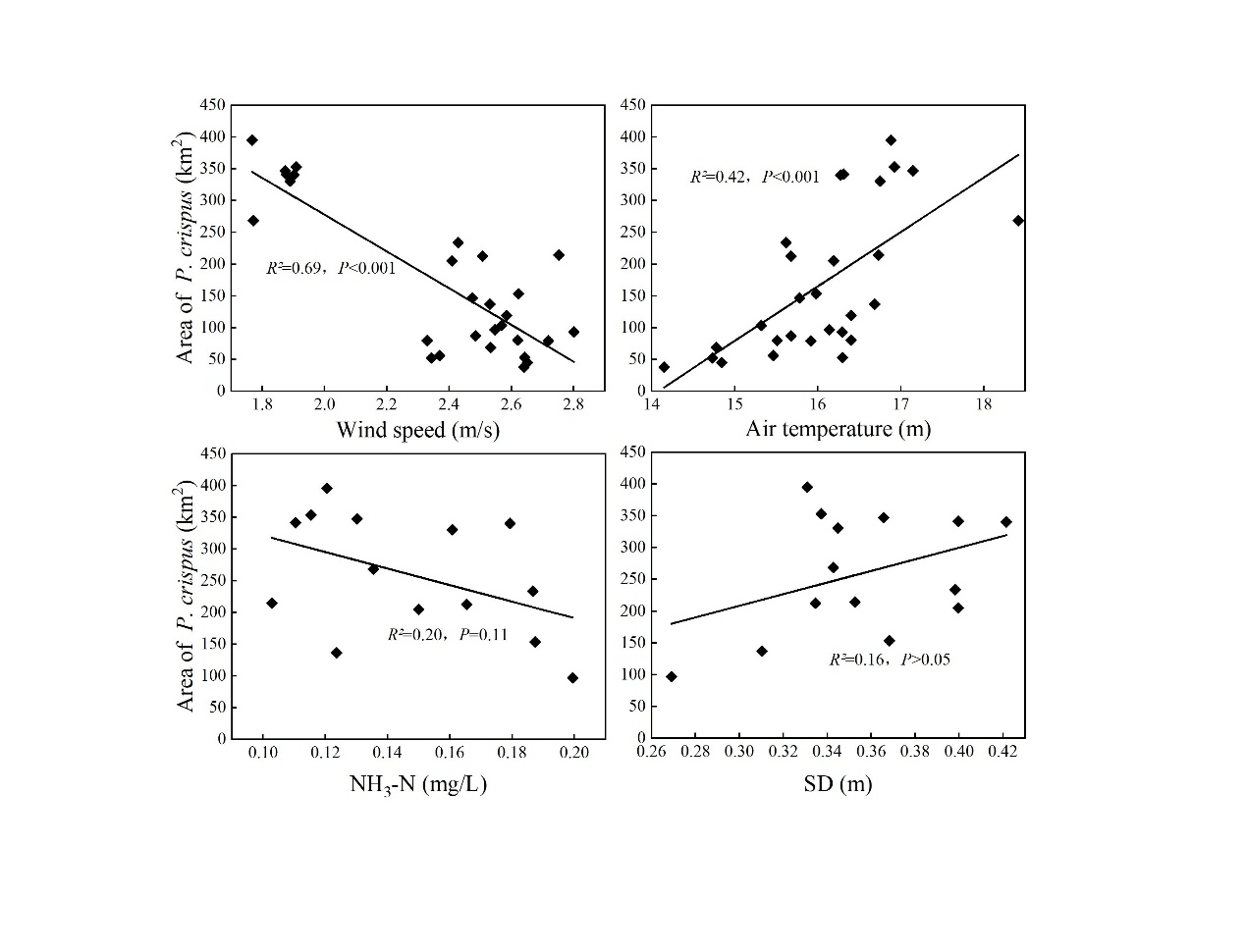
~~

**Supplementary Figure 8.** Linear fitting of the area of *P. crispus* and entire-year environmental factors


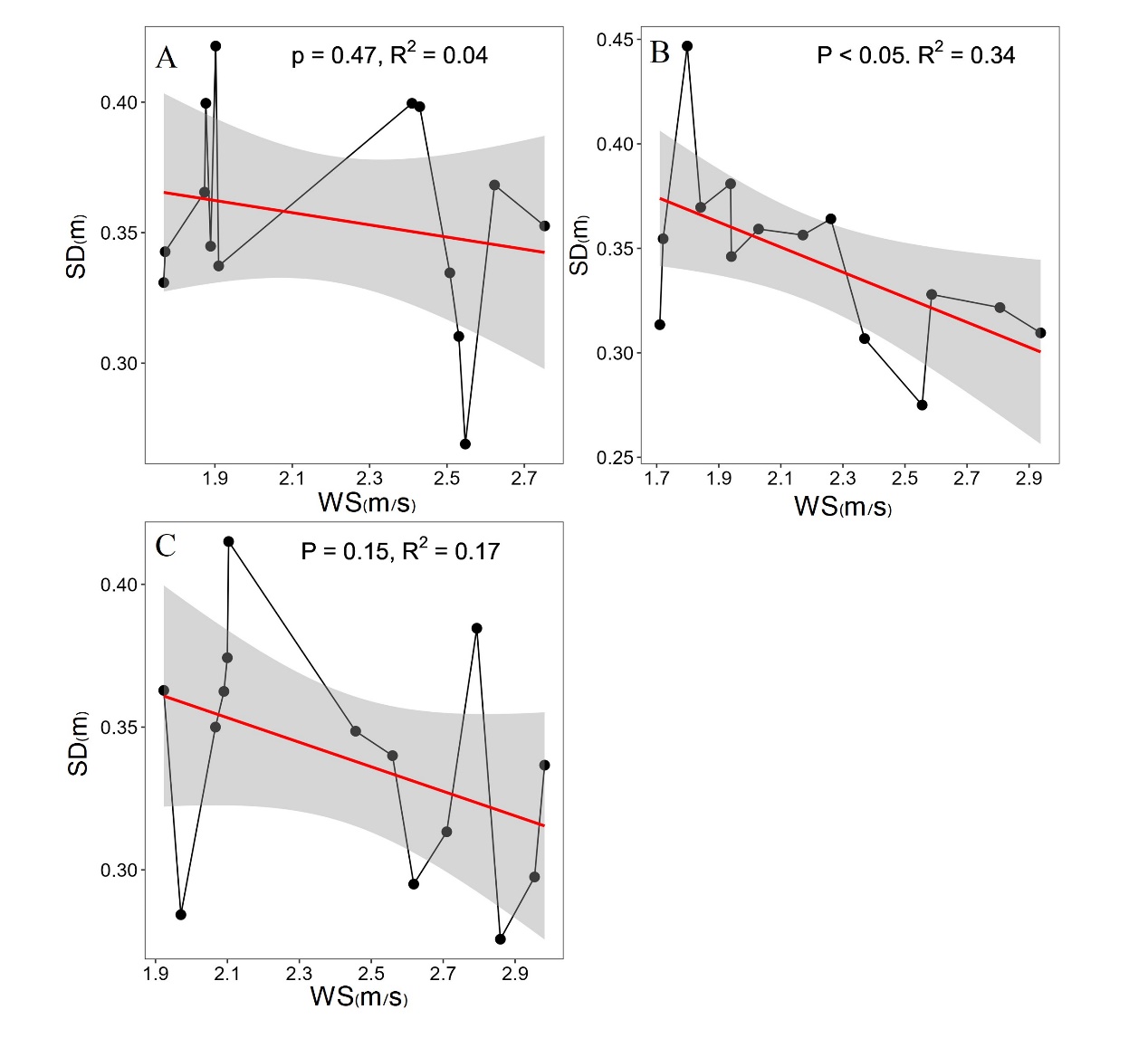


**Supplementary Figure 9.** Linear fitting of the WS and SD in (A) entire year, (B) winter and (C) spring.

**Table**

**Supplementary table 1**. Accuracy evaluation results of the classification

|  | | Measured class | | | | |
| --- | --- | --- | --- | --- | --- | --- |
|  | | FEM | Submerged Macrophyte | Water body | Sum | UA (%) |
| Mapped class | FEM | 65 | 4 | 1 | 70 | 92.86 |
|  | Submerged Macrophyte | 2 | 41 | 11 | 54 | 75.93 |
|  | Water body | 0 | 1 | 54 | 55 | 98.18 |
|  | Sum | 67 | 46 | 66 | 179 | / |
|  | PA (%) | 97.01 | 89.13 | 81.82 | / | / |
| OA =89%，Kappa =0.84 | |  | | | | |
